# PD31 - Pseudoallergic reactions to food and drugs in children with chronic idiopathic urticaria

**DOI:** 10.1186/2045-7022-4-S1-P31

**Published:** 2014-02-28

**Authors:** Ileana-Maria Ghiordanescu, Selda Ali, Roxana Silvia Bumbacea

**Affiliations:** 1 Elias Emergency University Hospital, Bucharest, Romania; 2 Regina Maria Clinic, Constanta, Romania; 3 Carol Davila University of Medicine and Pharmacy, Bucharest, Romania

## Introduction

Chronic idiopathic urticaria (CIU) is a condition in which histamine is constantly released from activated mastocytes and basophiles. A low degranulation threshold of mastocytes in response to different stimuli is described. Diamin oxidase defficiency can be found in patients with CIU. In children with CIU foods and drugs that interact with histamine metabolism can cause pseudoallergic reactions. For children few data in this field are available. Pseudoallergic reaction tends to appear at 4-6 hours after the ingestion of the culprit substance and are usualy not life-threatening.

## Material and methods

We present a case series of 4 children with CIU in which several urticarial episodes occurred in relation with ingestion of different foods and medication. The main causes for chronic urticaria were excluded. The possible culprit trigger was identified. Skin prick and intra-dermal tests were negative. Autologus Serum Skin was performed in 2 children. DAO serum activity and histamine plasma level were determined. An open challenge was performed 3 weeks after the elimination of the possible trigger, the avoidance of DAO inhibiting medication and the initialisation of a histamine-free diet.

## Results

The CIU diagnostic was confirmed in all cases. DAO activity was found to be low in all patients. Histamine plasma level was higher than normal. Provocation tests were positive in 2 patients. Avoidance methods had a positive effect in all cases.

## Conclusions

Pseudoallergic hypersensitivity reactions may be of relevance for chronic spontaneous urticaria with persistent symptoms. Patients can present urticarial episodes during treatments with molecules chemicaly not related. Usualy symptoms are non-reproducibles during provocation tests. We describe a case series of children with CIU and several exacerbations related to exposure to different foods and medication that had low DAO activity, high histamine plasma levels and improved after avoidance measures.

